# Chloroplast signaling within, between and beyond cells

**DOI:** 10.3389/fpls.2015.00781

**Published:** 2015-10-06

**Authors:** Krzysztof Bobik, Tessa M. Burch-Smith

**Affiliations:** Department of Biochemistry, Cellular and Molecular Biology, University of Tennessee, KnoxvilleTN, USA

**Keywords:** retrograde signaling, plastid signaling, redox, phytohormones, plasmodesmata, cell wall, stromules, stress responses

## Abstract

The most conspicuous function of plastids is the oxygenic photosynthesis of chloroplasts, yet plastids are super-factories that produce a plethora of compounds that are indispensable for proper plant physiology and development. Given their origins as free-living prokaryotes, it is not surprising that plastids possess their own genomes whose expression is essential to plastid function. This semi-autonomous character of plastids requires the existence of sophisticated regulatory mechanisms that provide reliable communication between them and other cellular compartments. Such intracellular signaling is necessary for coordinating whole-cell responses to constantly varying environmental cues and cellular metabolic needs. This is achieved by plastids acting as receivers and transmitters of specific signals that coordinate expression of the nuclear and plastid genomes according to particular needs. In this review we will consider the so-called retrograde signaling occurring between plastids and nuclei, and between plastids and other organelles. Another important role of the plastid we will discuss is the involvement of plastid signaling in biotic and abiotic stress that, in addition to influencing retrograde signaling, has direct effects on several cellular compartments including the cell wall. We will also review recent evidence pointing to an intriguing function of chloroplasts in regulating intercellular symplasmic transport. Finally, we consider an intriguing yet less widely known aspect of plant biology, chloroplast signaling from the perspective of the entire plant. Thus, accumulating evidence highlights that chloroplasts, with their complex signaling pathways, provide a mechanism for exquisite regulation of plant development, metabolism and responses to the environment. As chloroplast processes are targeted for engineering for improved productivity the effect of such modifications on chloroplast signaling will have to be carefully considered in order to avoid unintended consequences on plant growth and development.

## Introduction

According to the endosymbiotic theory plastids originated from free-living cyanobacteria that were engulfed by early eukaryotic cells. These cyanobacteria were retained by their hosts and have co-evolved with their host cells over 1.5 billion years to become an integral part of the modern plant cell (Yoon et al., 2004; Nakayama and Archibald, 2012). It is accepted that the successful stable integration occurred because of exceptional mutual benefits: the eukaryotic cell was able to establish an autotrophic lifestyle, while the engulfed cyanobacteria reached a pathogen-free asylum. Accumulating evidence suggests that this stable symbiosis between cyanobacteria and the eukaryotic cell was facilitated by infection of the latter with Chlamydiales pathogens (Ball et al., 2011, 2013).

The most conspicuous function of modern plastids is the sophisticated oxygenic photosynthesis performed by chloroplasts. However, plastids perform many other functions that are critical for proper plant development and physiology including the synthesis of amino acids, nucleotides and fatty acids, production of phytohormones, some vitamins and a multitude of secondary metabolites, as well nitrogen and sulfur assimilation. Many chloroplast secondary metabolites, besides being necessary for basic plant metabolic functions, are also important for interaction with the environment, as they function in plant defense against pathogen ingress and plant adaptation to stresses including heat, drought and high light. Thus, chloroplasts act as a hub in the cellular response to signals, generating a variety of signals that coordinate a fine-tuned and appropriate response to any given situation (Pfannschmidt and Yang, 2012).

**FIGURE 1:**
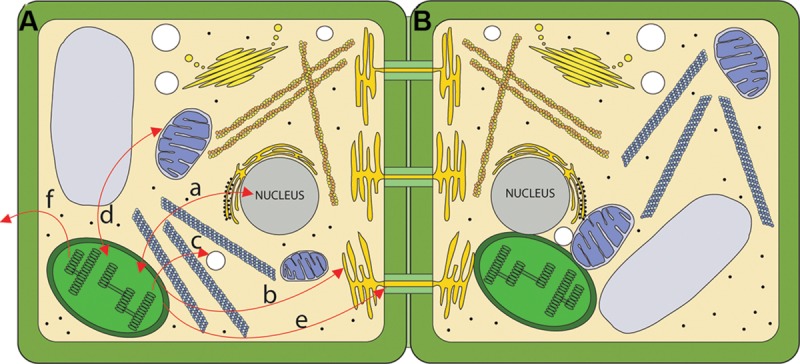
**Routes for chloroplast signaling. (A)** Chloroplasts generate signals that target multiple intercellular targets. (a) The majority of chloroplast proteins are encoded by the nucleus, and the import of those proteins into the chloroplast is anterograde signaling. In turn, several chloroplast products act as retrograde signals to regulate expression of nucleus-encoded genes. (b) Chloroplasts are metabolically coupled to the ER and it is likely that signals may move from the chloroplast to the ER. (c) Chloroplasts and peroxisomes are also closely associated, and numerous chloroplast products are substrates for peroxisomal pathways. (d) Mitochondria and chloroplasts are known to signal to each other. (e) Chloroplast signals regulate intercellular trafficking via plasmodesmata. It is not clear if this signaling is direct or involves retrograde signaling to the nucleus. (f) Chloroplasts produce volatile compounds that can signal to neighboring plants during pathogen attack. **(B)** The physical interaction between chloroplasts and various organelles may serve as a direct route for signaling.

The emerging view of the chloroplast is as a very dynamic signaling compartment. As a specific sensor of intra- and extracellular stimuli, chloroplasts constantly process and integrate a multitude of intracellular signals and pathways in order to sustain homeostasis at both the cellular and organismal levels (**Figure 1A**). An often over-looked aspect of cell biology is the physical interaction between organelles for coordination of signaling and metabolism (**Figure 1B**). This area is beginning to receive attention and we will examine these findings as they relate to chloroplasts and their roles in signaling. Given the dizzying array of signals that chloroplasts respond to and produce it is not surprising that there is considerable crosstalk between signaling pathways. This is particularly evident during responses to biotic and abiotic stress (Nakashima et al., 2014; Trotta et al., 2014; Zhou et al., 2015).

Chloroplast signaling is not limited to exerting its effects within the cell. It is clear that chloroplast-derived signals can travel far beyond their site of production to induce changes in distal parts of the plant (e.g., Petrillo et al., 2014). Further, chloroplasts apparently regulate intercellular trafficking via the channels known as plasmodesmata. Through this action, chloroplasts could regulate almost all aspects of plant growth and development as it is becoming clear that not only do metabolites but also hormones, transcription factors and small RNA molecules use these channels for intercellular communication. Beyond whole-plant signaling, chloroplast signaling can impact entire ecosystem through production of volatile compounds.

While we focus here on the chloroplasts in mature leaves, it can be expected that other types of plastids, e.g., proplastids, etioplasts, or leucoplasts, participate in signaling networks in response to their unique developmental states and environmental conditions. Further, it is also likely that at any given time different subpopulations of chloroplasts within a cell are in various metabolic or physiologic states and are therefore likely to be involved in distinct signaling processes. Thus chloroplast signaling is complex, and dissecting crosstalk and feedback mechanisms remains a daunting task. With attempts to engineer chloroplasts for specialized or improved metabolic outputs, attention must be paid to how these adjustments may impact chloroplast behavior if unintended consequences are to be diminished. These consequences would not be limited to the chloroplasts, but could extend even to other plants cultivated in the vicinity of the engineered plants.

## Chloroplasts in Intracellular Signaling

### Chloroplast Signaling to the Nucleus

Over evolutionary time a significant number of the cyanobacterial genes were transferred to the host nucleus (Race et al., 1999; McFadden, 2001). These genes subsequently acquired sequences that function as transit peptides to enable import of their protein products back into plastids. The nucleus therefore exerts considerable control over chloroplast functions, and this nucleus-to-chloroplast signaling is termed anterograde signaling. Importantly, several genes encoding proteins that are integral components of photophosphorylation or photosynthesis were retained in the cyanobacterial genome (Allen, 1993; Race et al., 1999; Raven and Allen, 2003). Thus, in order to establish a stable eukaryotic plant cell it was necessary to synchronize the activities of both genomes. This has been achieved by creating a complex signaling system between the nucleus and plastids, able to transfer information and efficiently adjust gene expression in both compartments according to particular needs. Plastid derived-signals that regulate nuclear gene expression represent retrograde signaling. While anterograde signaling is well understood, it has been more challenging to unravel the molecular details of retrograde signaling.

Identification of molecules and signaling strategies underlying so-called retrograde signaling represents a long-standing quest in plant biology. Historically, Bradbeer et al. (1979) provided the first report describing the existence of communication between those organelles. They observed that the barley (*Hordeum vulgare*) *albostrians* chloroplast ribosome-deficient mutant had severely decreased chloroplast protein synthesis, in conjunction with depressed expression of nucleus-encoded chloroplast genes. This revolutionary discovery was in opposition to Ellis’ “cytoplasmic control principle” that posited control of organellar protein synthesis by cytoplasmic components (Ellis, 1977). Bradbeer’s discovery was soon confirmed by other researchers who treated young seedlings of mustard, *Arabidopsis*, pea or barley with lincomycin, chloramphenicol or streptomycin – inhibitors of plastid protein synthesis (Oelmuller et al., 1986; Susek et al., 1993; Yoshida et al., 1998; Sullivan and Gray, 1999). Besides the strategy of relying on systems with compromised chloroplast ribosome function, other approaches to perturbing distinct aspects of chloroplast function have also successfully interrogated chloroplast-to-nucleus signaling. The induction of carotenoid deficiency in genetic mutants or in plants treated with norflurazon (a herbicide that inhibits phytoene desaturase and blocks carotenoid synthesis), as well as in plants with compromised tetrapyrrole synthesis (resulting in accumulation of intermediates) caused suppressed expression of nucleus-encoded chloroplast genes (Johanningmeier and Howell, 1984; Mayfield and Taylor, 1984; Susek et al., 1993; Kropat et al., 1997, 2000; Strand et al., 2003; Zhang et al., 2011). These and other studies led to the realization that the plastid functional status can regulate the expression of photosynthesis associated nuclear genes (PhANGs; Surpin et al., 2002). Moreover, analysis of the *Arabidopsis* chloroplast ribosomal protein mutant *rps1* revealed that chloroplast translational capacity is a critical factor in developing heat tolerance. This is mediated by inducing expression of the heat stress transcription factor HsfA2, a key regulator of heat tolerance (Yu et al., 2012). Therefore, the functional status of chloroplasts also regulates nuclear genes involved in heat-tolerance. The involvement of plastid translation in retrograde signaling and plant development was recently discussed in detail (Tiller and Bock, 2014).

The intense search for factors mediating this chloroplast-to-nucleus communication has identified a set of plastid metabolic intermediates. Importantly, proteins with functions in both chloroplasts and nuclei have been recently identified and have been proposed to participate in retrograde signaling. These retrograde signals are expected to work by modifying the expression of nuclear genes in order to adapt plant development and physiology to constantly changing environmental conditions. Currently two major modes of retrograde signaling are distinguished and they are involved in so-called biogenic and operational type of control. Whereas the former includes signals responsible for chloroplast and photosynthesis biogenesis, the latter act in response to changing environmental cues in fully developed chloroplasts (Pogson et al., 2008). The best-studied target of retrograde signaling is represented by PhANGs, but plastids are also involved in tuning the expression of nuclear genes involved in response to a plethora of biotic and abiotic conditions. A recent meta-analysis of microarray studies of systems where high levels of retrograde signaling were induced has identified a core module of 39 nuclear genes that were subject to regulation in response to all signals examined (Glasser et al., 2014). The genes in this group, presumably representing the core retrograde-response module, are all known to be responsive to sugar, reactive oxygen species (ROS), abscisic acid (ABA) and/or auxin signaling pathways. Thus retrograde signaling may exploit a common component of these signaling pathways to mediate changes in gene expression.

### The *GENOMES UNCOUPLED* (GUN) Mutants – Aiming to the Nucleus with Guns

Very helpful in deciphering the retrograde signaling phenomenon were mutants isolated from genetic screens. The *gun* (*genome uncoupled*) mutants escaped the pattern of suppressed PhANG expression despite defective chloroplast physiology or inhibited biogenesis. There are numerous excellent reviews of the role of *guns* in retrograde signaling (Woodson and Chory, 2008, 2012; Barajas-Lopez Jde et al., 2013; Chi et al., 2013). So far, six *gun* mutants have been identified and they can be classified according to pathways they belong to. Whereas the *gun1* mutant results from mutation in a gene encoding a chloroplast-localized pentatricopeptide repeat-containing protein (PPR), the *gun2-6* mutants are associated with tetrapyrroles synthesis (Susek et al., 1993; Mochizuki et al., 2001; Larkin et al., 2003; Strand et al., 2003; Koussevitzky et al., 2007). The exact role of GUN1 in PhANG regulation is far from understood, however, it is known to act upstream of ABSCISCIC ACID INSENSITIVE 4 (ABI4), an APETALA 2-type transcription factor that binds to the ACGT motif of light- and ABA-responsive elements (Koussevitzky et al., 2007). Interestingly, the expression of ABI4 was regulated by PTM, a chloroplast PHD-type transcription factor (Sun et al., 2011). The involvement of the key enzymes of the tetrapyrrole synthesis pathway in the *gun* phenotype led to detailed investigations of tetrapyrroles, especially Mg-protoIX, as putative retrograde signals. However no correlation between Mg-protoIX levels and retrograde signaling could be established (Matsui et al., 2008; Moulin et al., 2008). Interestingly, the *gun6* mutant identified heme as a strong candidate for mediating plastid-to-nucleus signaling (Woodson et al., 2011). Moreover, it was proposed that the impact of tetrapyrrole biosynthesis on nuclear gene expression is mediated by singlet oxygen (^1^O_2_)-induced signaling and feedback regulated 5-aminolevulinic acid (ALA) synthesis (Schlicke et al., 2014).

### SAL1-PAP Chloroplast Retrograde Pathway

**FIGURE 2:**
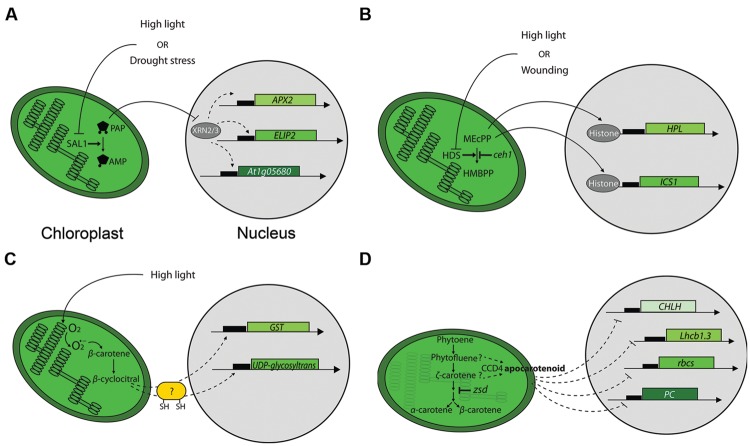
**Mechanisms of chloroplast-to-nucleus signaling. (A)** Retrograde signaling by PAP. High light or drought stress inhibits SAL1 phosphatase and leads to the accumulation of PAP. PAP likely inhibits specific exoribonucleases (XRNs) to modify nuclear genes expression. *APX2* and *ELIP2* stand for *ASCORBATE PEROXIDASE 2* and *EARLY LIGHT INDUCIBLE PROTEIN 2* genes, respectively. **(B)** Retrograde signaling by MEcPP. High light or wounding inhibits 1-hydroxy-2-methyl-2-(E)-butenyl-4-diphosphate synthase (HDS), leading to the subsequent accumulation of MEcPP. MEcPP affects nuclear gene expression via a mechanism proposed to involve chromatin remodeling by destabilizing DNA-histone interactions. *HPL* and *ICS1* stand for *HYDROPEROXIDE LYASE* and *ISOCHORISMATE SYNTHASE 1*genes, respectively. **(C)** Carotenoid-derivative β-cyclocitral mediates retrograde signaling. The ROS singlet oxygen induces formation of β-cyclocitral during high light treatment. β-cyclocitral’s action on selected nuclear genes is proposed to involve proteins containing sulphydryl groups. The genes depicted are *GLUTATHIONE-S-TRANSPHERASE (GST)* and *UDP-glycosyltransferase.* **(D)** An unidentified apocarotenoid affects expression of nuclear genes. It is proposed that the putative signaling apocarotenoid accumulates in chloroplasts due to compromised ζ-carotene desaturase activity that results in accumulation of phytofluene and ζ-carotene, putative substrates for the carotenoid cleavage deoxygenase 4 (CCD4) enzyme that is prerequisite for the putative apocarotenoid synthesis. *CHLH, Lhcb1.3, rbcs* and *PC* stand for genes encoding the subunit H of the Mg-chelatase complex, light-harvesting complex *1.3* isoform, the Rubisco small subunit and plastocyanin, respectively.

The detailed analysis of *sal1*, an *Arabidopsis* phosphonucleotidase mutant, has identified a known second messenger as acting in chloroplast-to-nucleus signaling. Estavillo et al. (2011) have demonstrated that the chloroplast and mitochondria-localized SAL1 phosphatase regulates the steady-state level of 3′-phosphoadenosine 5′-phosphate (PAP) by dephosphorylating it to an adenosine monophosphate (AMP). In the *sal1* mutant, or in response to drought stress or high light intensity, PAP levels increased, inducing expression of *ASCORBATE PEROXIDASE 2* and *EARLY LIGHT INDUCIBLE PROTEIN 2*, two nuclear genes whose expression is induced by high light stress (Harari-Steinberg et al., 2001; Caverzan et al., 2012). It has been proposed that PAP travels from chloroplasts to the nucleus where it regulates nuclear gene expression. Nucleus-localized exoribonucleases (XRNs) are likely targets of PAP, and by repressing their activity PAP may stimulate expression of high light and drought-responsive genes, leading to increased tolerance (Estavillo et al., 2011; **Figure 2A**).

### Methylerythritol (MEcPP) Retrograde Pathway

Isoprenoid metabolism is a major biosynthetic pathway in plants (Cordoba et al., 2009). The *Arabidopsis constitutively expressing HPL (ceh1*) mutant displays enhanced expression of *hydroperoxide lyase (HPL)*, a stress-inducible nuclear gene encoding a plastid-localized protein of the oxylipin pathway. The *ceh1* mutation disrupted a plastid-localized enzyme (HDS) that catalyzes conversion of methylerythritol cyclodiphosphate (MEcPP) to hydroxymethylbutenyl diphosphate (HMBPP; Xiao et al., 2012). The absence of CEH1 led to accumulation of MEcPP and induced the expression of a subset of stress-associated genes, including *ISOCHORISMATE SYNTHASE 1* (a key plastidial enzyme in the salicylic acid (SA)-biosynthetic pathway) and *HPL*, but not *ALLEN OXIDE SYNTHASE* [*AOS*, encoding a plastid-localized protein of the jasmonic acid (JA)-biosynthetic pathway]. HDS-depleted plants with increased levels of MEcPP accumulated SA and displayed increased resistance to infection by biotrophic pathogens. Importantly, elevated MEcPP levels and increased expression of *HPL* are observed upon both wounding and high light treatment, demonstrating involvement of MEcPP in a retrograde pathway involved in abiotic stresses (operational control) distinct from the *gun* signaling pathway. Therefore MEcPP is a retrograde signal inducing targeted stress responses. The proposed mechanism of MEcPP action involves direct modification of chromatin remodeling by disruption of DNA-histone interactions (Xiao et al., 2012; **Figure 2B**).

### Derivatives of Carotenoids as Signaling Molecules

Carotenoids are tetraterpenoid products of the isoprenoid biosynthetic pathway that also generates ABA and strigolactones (Ruyter-Spira et al., 2013; Giuliano, 2014). Carotenoids are constituents of the light harvesting complexes where they serve as accessory pigments to extend the absorption spectra of the chlorophylls, and they have critical protective roles as scavengers of singlet oxygen (^1^O_2_) generated by the PSII reaction center (Telfer, 2014). Carotenoid derivatives have recently been proposed to act as chloroplast-generated signaling molecules that link chloroplast activity and nuclear gene expression (Ramel et al., 2012; Avendano-Vazquez et al., 2014; Van Norman et al., 2014).

β-cyclocitral is a product of singlet oxygen-induced β-carotene oxidation. This volatile molecule contains an α,β-unsaturated carbonyl, designating it as a reactive electrophile species (RES). β-cyclocitral’s accumulation in *Arabidopsis* leaves during high-light stress correlated with accumulation of singlet oxygen, supporting the notion of β-cyclocitral as an oxidation product of β-carotene (Ramel et al., 2012). Consistent with this, transcriptome analysis by DNA microarrays revealed that about 80% of β-cyclocitral-induced or repressed genes are also responsive to singlet oxygen overproduction in the *Arabidopsis fluorescent (flu)* mutant (op den Camp et al., 2003). Using qRT-PCR Ramel and coworkers demonstrated that all singlet oxygen marker genes tested are also induced by β-cyclocitral. Importantly, this effect seemed to be specific to β-cyclocitral as the overlap between gene expression changes induced by β-cyclocitral and other RES like methyl vinyl ketone or malondialdehyde (MDA) was smaller. Among the genes most strongly induced by β-cyclocitral were 10 glutathione-*S*-transferase (GST) genes and 12 UDP-glycosyltransferases (**Figure 2C**). Both groups of genes are involved in detoxification processes conferring tolerance to singlet oxygen in *Chlaydomonas reinhardtii* (Ledford et al., 2007). The protective effect of β-cyclocitral resulted in better PSII quantum efficiency and lower lipid peroxidation under high light stress (Ramel et al., 2012). Finally, the current model proposes β-cyclocitral as a stress molecule generated in chloroplasts under photooxidative stress that reprograms gene expression leading to stress acclimation. Thus, in addition to their roles in light harvesting and as antioxidants, carotenoids can also act as signaling molecules. The exact mechanism of β-cyclocitral action or its receptor in the nucleus are unknown, however, it is proposed that as an electrophile with a α,β-unsaturated carbonyl group it could react with electron donors such as proteins containing sulphydryl groups (Ramel et al., 2012, 2013). Very recently, elevated levels of β-cyclocitral were reported in *Arabidopsis* plastoglobule kinase mutants that have defective plastoglobule metabolism (Lundquist et al., 2013). The *abc1k1 abc1k3* double mutant shows rapid chlorosis under high light stress, confirming β-cyclocitral’s, role in mediating stress responses.

In addition to β-cyclocitral, apocarotenoids are also potential chloroplast retrograde signaling components. Analysis of the *Arabidopsis ζ-carotene desaturase* mutant (*zds/clb5/spc1/pde181*) displaying arrested chloroplast biogenesis at a very early stage of development led to the conclusion that the accumulation of an uncharacterized apocarotenoid can act as a retrograde signal (Avendano-Vazquez et al., 2014). This apocarotenoid is likely generated by the activity of the carotenoid cleavage deoxygenase 4 (CCD4) enzyme on ζ-carotene (**Figure 2D**). Accumulation of this putative cleavage product was shown to modulate expression of many nuclear genes required for leaf development leading to a severe phenotype that included arrested chloroplast development and leaves with defective adaxial-abaxial patterning. Since neither ROS nor ABA nor strigolactone signaling pathways were responsible for the observed phenotypes, it was concluded that the putative phytofluene or ζ-carotene –derived apocaroteniod is part of a novel retrograde signaling pathway. Interestingly, the observed defects were restricted to primary leaves, underscoring the differences in developmental regulation between plastids in different organs (Avendano-Vazquez et al., 2014).

A third carotenoid derivative is implicated in plastid retrograde signaling, this time with respect to lateral root (LR) development (Van Norman et al., 2014). Reduced LR formation in *Arabidopsis* seedlings treated with norflurazon was observed and further investigation indicated that a β-carotene derivative is required for prebranch site formation (Van Norman et al., 2014). Extensive genetic analyses ruled out ABA and strigolactone as the carotene-derived signaling molecule involved in LR formation. Additionally, treatment of *Arabidopsis* seedlings with D15, a candidate inhibitor of carotenoid cleavage at the 9,10 position, resulted in a highly significant decrease in LR capacity, suggesting that this unknown apocarotenoid is likely cleaved at this position. Interestingly, even though reduced LR capacity and small albino shoots were observed in carotenoid biosynthesis mutants and in plants treated with norflurazon, plants treated with D15 had green shoots of comparable size to wild type. This demonstrates that the involvement of carotenoids in LR formation is separate from their photoprotective function (Van Norman et al., 2014). Intriguingly, carotenoid biosynthesis was found to occur in differentiated parts of the root at some distance from the oscillating zone where prebranch sites and eventually LRs formed. Thus the non-cell autonomous function of (apo)carotenoids seems to be required for development of LRs (Van Norman et al., 2014).

### Chloroplast Proteins as Retrograde Signals

It is a broadly accepted paradigm that most nucleus-encoded chloroplast proteins reach this compartment due to the transit peptides located at their N-termini. Cleavage of the signal peptides after entry in to the chloroplast generates the functional chloroplast proteins. However, accumulating data show that some chloroplast proteins also act in the nucleus. While it is tempting to speculate that this is attributable to two-way protein movement between chloroplasts and cytoplasm, this has not been unambiguously demonstrated. However, a physiological function for this chloroplast-nucleus dual localization is apparent and it seems to be indispensable for proper plant response to pathogen attack and abiotic stress, and emphasizes the function of chloroplasts as signaling compartments.

**FIGURE 3:**
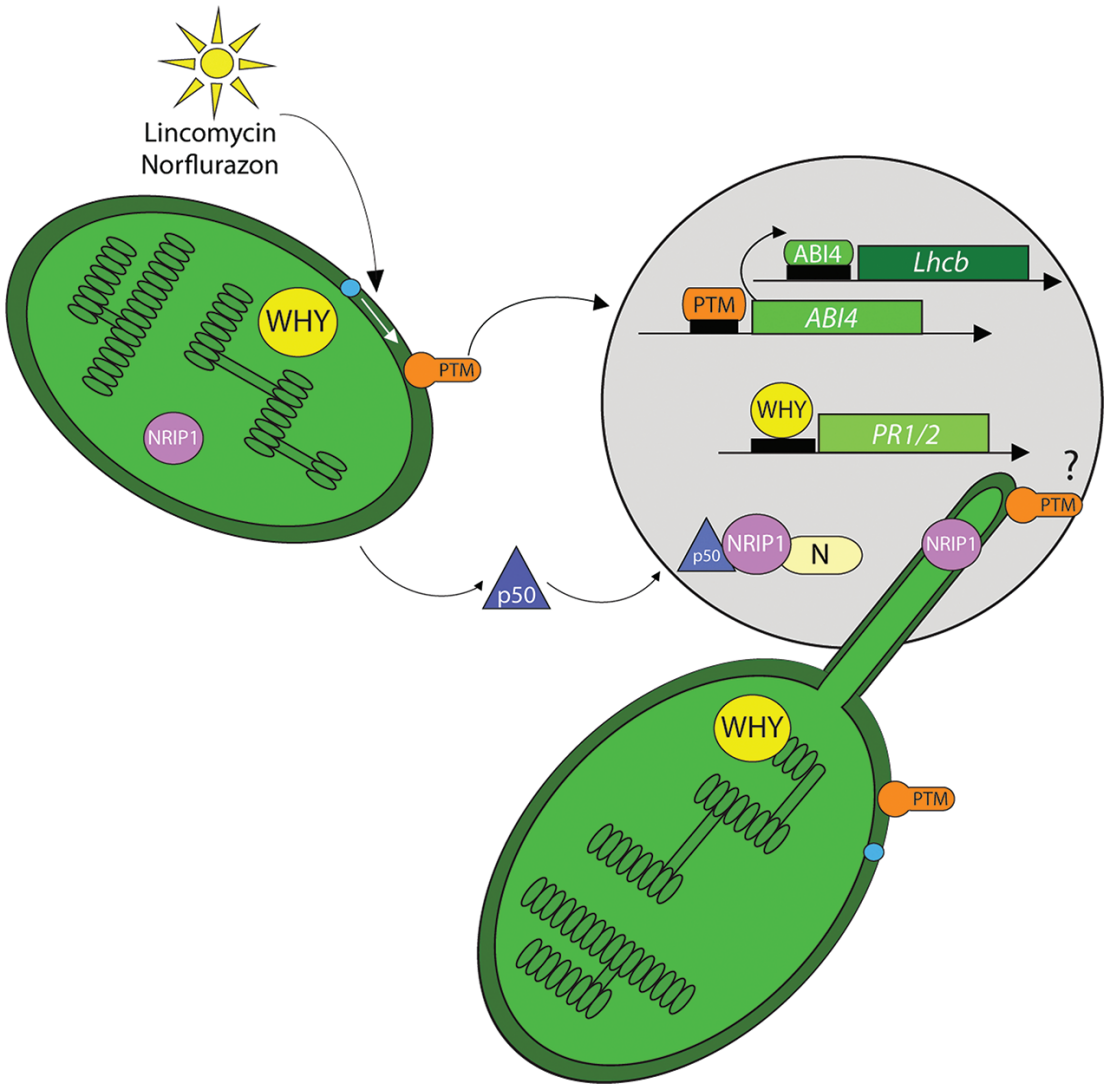
**Chloroplast proteins as retrograde signals.** A few chloroplast proteins have been implicated in directly modulating nuclear gene expression by their nuclear localization. These proteins may transit the cytoplasm by an unknown mechanism. Alternatively, it has also been proposed that they may move through stromules to enter the nucleus. High light, lincomycin or norflurazon treatments induce a serine protease-dependent (blue dot) proteolytic-cleavage of the PTM, a chloroplast envelope-bound plant homeodomain (PHD) transcription factor. The cleavage product is found in the nucleus where it binds to promoter region of the ABI4 transcription factor. ABI4 in turn associates with the regulatory sequences of the *Lhcb* genes and prevents their transcription. The chloroplast protein Whirly1 also localizes to the nucleus and this is correlated with increased expression of *PATHOGENESIS RELATED GENE 1* and *2* (*PR1/2*). Upon TMV infection the chloroplast-localized NRIP1 is also detected in the nucleus where it interacts with the helicase domain of the TMV replicase (p50). Finally a trimeric complex of p50, NRIP1 and the N protein is localized to nucleus to provide resistance against the virus. It is suggested that NRIP1 may use stromules to translocate from the chloroplast to the nucleus.

One such protein, PTM (PHD type transcription factor with transmembrane domains) was shown to provide a physical link in signaling between chloroplasts and nucleus to regulate gene expression (Sun et al., 2011). This membrane-bound transcription factor (MTF) is localized to chloroplast outer envelope by four transmembrane domains at its C-terminus (**Figure 3**). The N-terminus of PTM contains a DNA-binding homeodomain box, a different transcription factors (DDT) domain and a plant homeodomain (PHD). Interestingly, a shorter variant of this protein, lacking the transmembrane domains, was detected in nuclear fractions. Notably, increased amounts of the shorter PTM variant were detected upon treatment with either norflurazon or lincomycin, and on exposure to high light. Through the application of protease inhibitors, it was demonstrated that the shorter form of PTM was the result of serine protease activity (Sun et al., 2011; Adam, 2015). According to the proposed model, chloroplast signals induce the intramembrane proteolytic cleavage of full length PTM, producing a soluble shorter variant (∼58 kDa) that is released to the cytoplasm and finally transclocates to the nucleus where it binds, through its PHD domain, to the *ABI4* promoter to induce *ABI4* expression. ABI4, in turn binds to the *Lhcb* promoter, close to the CUF1 element and precludes binding of G-box-binding factors required for the expression of *Lhcb* and other PhANGs. This model explains the *gun* phenotype observed in *ptm* and *abi4* mutants. Moreover, the amount of processed PTM declined in the *gun1* mutant, suggesting a complex regulatory network.

WHIRLY1 provides another example of a chloroplast protein with a role in the nucleus. A WHIRLY1 fusion protein expressed in the plastid genome of tobacco, localized to both plastids and nuclei, and the two subpopulations were the same molecular size (Isemer et al., 2012). As a consequence WHIRLY1-regulated *PR* (*PATHOGENESIS RELATED*) genes were upregulated under normal growth conditions (**Figure 3**). WHIRLY1 is part of the transcriptionally active chromosome in plastids, and interestingly, another component of this complex, pTAC12 (HEMERA), also showed dual localization to chloroplasts and nuclei with unchanged molecular mass, excluding any proteolytic modification (Chen et al., 2010). However, unlike in the case of WHIRLY1, the dual localization of HEMERA in a single cell has not yet been demonstrated. Such a demonstration is important for ruling out the possibility that dual localization could be caused by fluctuating distribution resulting from specific cell types or developmental stages. It has been proposed that WHIRLY1 conveys information about the chloroplast redox state to the nucleus, and SA regulates this communication (Foyer et al., 2014). The proposed mechanism of WHIRLY1 action would be similar to that of NPR1 (See below). The mechanism and consequences of the proposed translocation of NRIP1 (another chloroplast-localized protein) to the nucleus is discussed later.

There are a few hypotheses that aim to explain the nuclear localization of chloroplast-targeted proteins and they invoke mechanisms enabling translocation of chloroplast-localized proteins to nuclei. One of them proposes permeabilization of the chloroplast outer membrane by an unknown mechanism. Another posits that stress-induced modification of chloroplasts results in the formation of stromules that contact nuclei could facilitate direct trafficking of chloroplast proteins (Caplan et al., 2008). In agreement with the former, a recent communication described GFP-fusion protein leakage from functional chloroplasts upon pathogen attack (Kwon et al., 2013). Interestingly, this occurrence was shown to be dependent on ROS. Evidence for the latter hypothesis of stromule involvement in chloroplast protein translocation is now being reported (see Stromules below). Regardless of the mechanism governing the distribution of chloroplast proteins to the cytoplasm and/or nucleus, this phenomenon may represent an important pathway for direct communication between chloroplasts and nuclei, and provides important insights in understanding the molecular basis of retrograde signaling.

## Chloroplasts and Inter-Organellar Signaling

**FIGURE 4:**
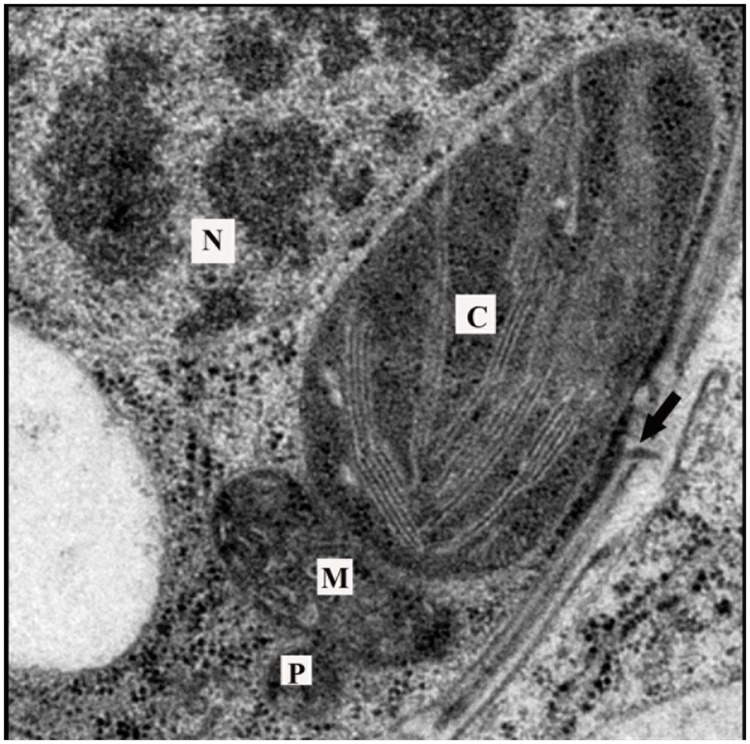
**Arrangement of organelles in a leaf cell.** Transmission electron microscopy images often reveal chloroplasts in close proximity with peroxisomes and mitochondria. Chloroplasts can also be observed near the nucleus and cell wall. Note the presence of a plasmodesma in the cell wall (arrow). Such arrangements of organelles would minimize distances that signals must traverse to arrive at their target. C, chloroplast; M, mitochondria; P, peroxisome; N, nucleus.

It has long been appreciated that there is metabolic crosstalk between organelles. Metabolic pathways often involve multiple organelles and metabolic intermediates may be transported across membranes by diffusion or by specialized transporters in an energy-dependent manner after traversing the cytosol. However, there is emerging evidence that direct physical contact between organelles may provide a major route for metabolic exchange (**Figure 4**). Such direct contact would also provide routes for inter-organelle signaling, although evidence for this is still limited. Thus, chloroplasts, besides producing signals that may travel long distances from plastids to their targets, may also communicate directly with other organelles through physical contacts. Here we consider evidence for chloroplast-organelle contacts and possible roles for these contacts in signaling.

### Stromules for Plastid-to-Plastid and Plastid-to-Nucleus Signaling

Rediscovered by Kohler et al. (1997), the observation of tubular protrusions from plastids changed our thinking about chloroplasts and possible pathways for signaling (Kohler et al., 1997). These so-called stromules are stroma-filled tubules enclosed by the inner and outer plastid envelope membranes, and are 0.4–0.8 μm in diameter and of variable length typically up to 65 μm (Gray et al., 2001). They are more abundant in non-green plastids than in chloroplasts. Stromules are distinct from the chloroplast protrusions (CPs) that form during stress (Holzinger et al., 2007a,b), and that are involved in the sequestration of Rubisco from the rest of the chloroplast body (Yamane et al., 2012).

Stromule formation is dependent on both intrinsic and extrinsic factors. The size of plastid, plastid identity, state of differentiation and density of plastids all determine stromule formation (Waters et al., 2004). The actin cytoskeleton has also been reported to be important for stromule formation and movement (Kwok and Hanson, 2003; Kwok and Hanson, 2004b; Gunning, 2005; Holzinger et al., 2007b), and the myosin XI motor is required for stromule formation in *Nicotiana benthamiana* chloroplasts (Natesan et al., 2009; Sattarzadeh et al., 2009). In contrast, it has recently been reported that isolated chloroplasts can form stromules (Brunkard et al., 2015b). Stromule formation is also temperature sensitive, and temperatures around 20°C appear optimal for stromule formation (Holzinger et al., 2007a), while lower temperatures inhibit their formation (Gray et al., 2012) and higher temperatures induce CP formation (Holzinger et al., 2007a). The light-dependence of stromule formation is somewhat controversial. There have been reports that light is not required for stromule formation (Kwok and Hanson, 2003; Gray et al., 2012), but recent findings demonstrate increased formation of stromules from *Arabidopsis* mesophyll chloroplasts during the day as compared to the night (Brunkard et al., 2015b). It is possible the differing results can be explained by differences in plant growth conditions and the types of plastids examined in each experiment. Besides light and temperature the hormone ABA, likely generated in response to environmental stresses, also induces stromule formation (Gray et al., 2012). Consistent with this, salt and osmotic stress also increased the fraction of stromule-bearing chloroplasts.

The most obvious consequences of stromules are significant enlargement of both plastid envelope surface and plastid volume. These modified plastid properties could affect the rate of plastid import and export, and also cause changes in plastid compartmentalization, respectively. Consistent with these presumed functions, it was shown that chlorophyll and thylakoid membranes are absent from stromules (Kohler et al., 1997; Holzinger et al., 2007a; Newell et al., 2012). The trafficking of plastid genomes or genetic material via stromules has also been ruled out (Newell et al., 2012). On the other hand proteins like GFP, aspartate aminotransferase and Rubisco complexes of a molecular weight around 550 kDa have been localized within stromules (Kwok and Hanson, 2004a). Moreover, interconnections between individual plastids via stromules have been reported, and photobleaching experiments and the use of photoconvertible proteins have demonstrated the transfer of proteins between them (Kohler et al., 2000; Hanson and Sattarzadeh, 2011, 2013, but see Schattat et al., 2012, 2015). Given that connections between plastids are rare, the biological significance of possible plastid-to-plastid trafficking remains unclear (Hanson and Sattarzadeh, 2013).

Stromules are involved in metabolic responses to stress, namely chloroplast autophagy in response to nutrient starvation (Ishida et al., 2008). Through observations of fluorescently tagged proteins and the *Arabidopsis atg5* autophagy mutant, it was demonstrated that plastid stromal proteins can be remobilized to the vacuole via the ATG-dependent authophagic pathway, without destroying the chloroplast. Similar observations have been reported from rice (Izumi et al., 2015). According to the proposed model stressed-induced autophagy sequesters stromules by forming an isolation membrane that eventually clips off a given stromule and its stromal contents, and then transports the cargo to the vacuole for degradation (Ishida et al., 2008). Interestingly, both stromules and protrusions were identified in potato tuber amyloplasts (Borucki et al., 2015). These protrusions, unlike stromules, are likely involved in starch accumulation in the parenchyma storage cells. The involvement of protrusions in accumulation of starch had been previously demonstrated (Langeveld et al., 2000). Thus stromules are involved in metabolism.

Plastids and stromules have been repeatedly observed in close proximity to other organelles including mitochondria, other plastids, ER, plasma membrane and nuclei. From the perspective of signaling, however, the most interesting seems to be the distinct distribution of plastids around nuclei, including concentration of stromules around and, most intriguingly, inside the nucleus (Collings et al., 2000; Kwok and Hanson, 2004c). Clusters of plastids with long stromules of 20–30 μm localized around the nucleus were observed in *N. tabacum* petioles of cotyledons (Kwok and Hanson, 2004c), as well as in the lower part of the hypocotyl where the plastids were preferentially arranged around the nucleus with long stromules of up to 100 μm extending to the cell periphery (Natesan et al., 2005). Such concentration of plastids around nucleus was also observed in petal cells and in shoot meristems (Kohler and Hanson, 2000; Kwok and Hanson, 2004c). Stromules have also been observed lying in grooves and invaginations of the nuclear membranes in tobacco epidermal cells (Kwok and Hanson, 2004c). Such direct connections were proposed to increase efficiency in plastid-nucleus communication. Stromules have also been implicated in chloroplast-to-ER signaling (Schattat et al., 2011a,b), and in intercellular signaling (Kwok and Hanson, 2004c) but this has not been explicitly tested or proven. These suggestions are based on interaction between stromules and ER or the plasma membrane, respectively, and further characterization of these interactions is warranted.

Stromules and stromule-nucleus contacts may have important roles in host–pathogen interactions. The induction of stromules and remobilization of chloroplasts to surround nuclei was observed in *N. benthamiana* leaves in response to infiltration with GV3101, common lab strain of *Agrobacterium tumefaciens* (Erickson et al., 2014). In addition, starch accumulated in GV3101-treated leaves and the levels of soluble sugars also increased. However, another lab strain LBA4404 did not produce these effects. The introduction of the *trans zeatin synthase* (*tsz*) gene from the GV3101 plasmid into LBA4404 led to the induction of stromules and other cellular changes typically observed on GV3101 infiltration. Indeed, direct application of cytokinin to leaves produced phenotypes similar to those obtained with GV3101. Thus, cytokinin may mediate the production of stromules and chloroplast movements during some plant–pathogen interactions.

**FIGURE 5:**
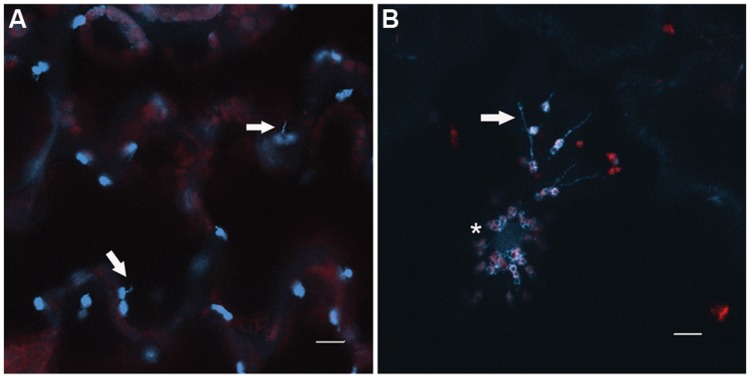
**Chloroplast behavior during defense. (A)** Stromules (arrows) are observed intermittently from chloroplasts in the epidermis of *Nicotiana benthamiana* leaves. **(B)** Upon infection with Tobacco mosaic virus, chloroplasts cluster around the nucleus (asterisk), and stromule formation is induced (arrow). The chloroplasts shown are expressing NRIP1-CFP (Caplan et al., 2008, 2015). Note the NRIP1-CFP signal detected in the nucleus in TMV-infected chloroplasts, indicative of the translocation of NRIP1 from the chloroplasts to the nucleus. Images were collected on a Zeiss LSM 710 confocal laser scanning microscope and single focal plane images are shown. Scale bar is 10 μm.

The question of why stromule induction and chloroplast-nucleus associations occur during plant–pathogen interactions has recently been addressed. *Nicotiana* N RECEPTOR INTERACTING PROTEIN (NRIP)1 is a chloroplast rhodanese sulphurtransferase that is required for effector triggered immunity (ETI) against TMV mediated by the N innate immune receptor (Caplan et al., 2008). In the presence of the viral p50 helicase effector NRIP1 was recruited to the cytoplasm and nucleus where it formed a protein complex with the TIR domain of the N immune receptor (**Figure 3**). The interaction with N in the nucleus and cytoplasm is necessary for ETI. These results raised the intriguing questions of whether NRIP1was remobilized from the chloroplast and how this could be accomplished. Recent data suggest that stromules may provide a route for NRIP to traffic from the chloroplast to the nucleus. Co-expression of N and p50 induced formation of stroumles during HR-PCD associated with this interaction (Caplan et al., 2015; **Figure 5**). Similarly, stromule induction was observed during ETI initiated in response to bacterial pathogens, and on treatment with the defense-related signaling molecules SA and the ROS H_2_O_2_. Through correlative EM-fluorescence microscopy, the authors provide convincing evidence that stromules contact the nucleus during N-mediated defense. In elegant experiments that fuse a nuclear exclusion sequence (NES) to NRIP, the authors provide quantitative evidence that NRIP1 is indeed trafficked from the chloroplast to the nucleus. H_2_O_2_ also traffics to the nucleus via stromules. In plants overexpressing chloroplast outer membrane protein CHLOROPLAST UNUSUAL POSITIONING 1 (CHUP1) stromule formation is abolished, suggesting that this membrane plays a role in stromule formation. This was confirmed in *chup* mutants and knockdown plants, where constitutive stromule formation was observed. The importance of stromules to HR-PCD and the defense response was underscored by the accelerated HR-PCD observed in those plants. These results are suggestive of a role for stromules in intracellular trafficking and possibly signaling during plant–pathogen interactions, and these possibilities warrant further examination. It will be exciting to test whether plastids and/or stromules are able to create hemifusion membranes with the nuclear membrane and whether other signaling molecules or metabolites use this route for chloroplast signaling.

A separate study raises the possibility that stromules may also function in intercellular and intracellular trafficking of pathogens. The chloroplast-localized chaperone heat shock cognate 70 kDa protein (cpHsc70-1) was identified as interacting with the AbMV movement protein (MP; Krenz et al., 2010). In uninfected tissues cpHsc70-1-YFP homooligomerization was demonstrated by bimolecular fluorescence complementation (BiFC). cpHsc70-1-YFP oligomers localized to chloroplasts and near the cell periphery. Intriguingly, cpHsc70-1-YFP localized to punctate structures in chloroplasts and filaments that stretched like a “string of pearls” between chloroplasts or to the cell periphery in AbMV-infected leaves. Reduced levels of the Hsc70 ortholog in *N. benthamiana* by virus induced gene silencing (VIGS) led to decreased intercellular trafficking of AbMV while viral replication was unaffected, suggesting that the chloroplast chaperone and stromules may have a role in viral trafficking that is independent of viral replication and accumulation (Krenz et al., 2010). Indeed, it has been proposed that the Hsc-70 chaperone in stromules may facilitate viral transport from chloroplasts to the cell periphery and plasmodesmata, from where intercellular spread could occur (Krenz et al., 2012). AbMV is a geminivirus that replicates in the plant nucleus, but AbMV is also found in plastids (Groning et al., 1987); therefore stromule-nucleus contacts could also provide a route for viral trafficking to nuclei in newly infected cells.

Another interesting question regarding the involvement of stromules in chloroplast signaling is when during evolution would such an innovation have arisen. The R gene *RESISTANCE TO POWDERY MILDEW* (*RPW)8.2* confers broad resistance to fungi that cause powdery mildew. RPW8.2 localizes to the extrahaustorial membrane (EHM) during fungal penetration of the host cell (Wang et al., 2009). Interestingly, RPW8.2 mutants have recently been reported to localize to an unknown membrane that appears to surround stromules (Wang et al., 2013a). This membrane has been termed the peristromule membrane (PSM) and it is proposed that they may share some as-yet-unknown characteristics with the EHM. Some RPW8.2 mutants localize to both the EHM and PSM, and while others localize to the nucleus. Notably, invasion by fungal haustoria induced the formation of stromules that purportedly connect plastids with haustoria. The authors speculate that stromules may represent an ancient interface for host–pathogen interactions. However, wild-type RPW8.2 was not observed labeling the PSM, and the authors speculate that this may be due to rapid cycling of RPW8.2 on and of the PSM (Wang et al., 2013a).

Together, these studies suggest that stromules could function in signaling in mediating responses to both biotic and abiotic stress, however, this remains to be demonstrated. Understanding the cellular functions of stromules will no doubt reveal intriguing aspects of plant cell biology and possibly identify new targets for engineering plants with modified responses to biotic and abiotic stresses.

### Chloroplast-Endoplasmic Reticulum Contacts: Lipid Signaling

A conundrum in plant biology is that the enzymes for a given metabolic pathway are often found in different subcellular compartments. One striking example of this is the biogenesis of complex lipids in some plants including *Arabidopsis*. In these plants fatty acids synthesized *de novo* in the chloroplast by the prokaryotic pathway may be exported to the ER where they are assembled into lipids by the eukaryotic pathway before import back into chloroplasts (Benning, 2009). Despite concerted efforts to identify candidate transporters that would allow the substrates to translocate from the chloroplast to the ER, these transporters have not yet been discovered (Wang and Benning, 2012). This raises the intriguing possibility that metabolites may traffic directly from the chloroplast to the ER through membrane interactions. Moreover, direct physical contact sites between chloroplasts and ER, named plastid-associated membranes (PLAM), have long been reported by transmission electron microscopy in many plant and algal species (Cran and Dyer, 1973; Crotty and Ledbetter, 1973; Renaudin and Capdepon, 1977; McLean et al., 1988). More recently, confocal microscopy has also suggested chloroplast-ER membrane contact sites (MCS; Andersson et al., 2007; Tan et al., 2011). Indeed, these MCS are likely held together through strong protein–protein interactions since the ER remained associated with chloroplasts even after application of forces of 400 pN (Andersson et al., 2007). Once synthesized in the ER glycerolipids have to be imported into the chloroplasts. Mathematical modeling supports the import of diacylglycerol (DAG) from the ER to chloroplasts (Marechal and Bastien, 2014). Genetic analyses have identified the TGD complex as essential for import of ‘eukaryotic’ precursors into chloroplasts (Boudiere et al., 2014). TGD1-3 constitute a bacterial ABC transporter, while TGD4 apparently forms a β-barrel that localizes to both chloroplasts and ER (Wang and Benning, 2012). Thus transporters are indeed important mediators of lipid trafficking.

The DellaPena group has proposed an elegant hypothesis to explain chloroplast-ER membrane continuity (Mehrshahi et al., 2014). This group has developed a transorganellar assay to test whether non-polar metabolites, exemplified by tocopherols (vitamin E), located in the plastid envelope could directly access the lumen of the ER and modulate enzymes located there (Mehrshahi et al., 2013). To do this, tocopherol cyclase (TC) that is normally a chloroplast resident protein was retargeted to the ER in the background of the *vte1* mutant that does not make TC and tocopherols (Porfirova et al., 2002). Excitingly, this ER-localized TC complemented the *vte1* mutation and tocopherol levels were restored to almost wild-type levels in the rescued lines (Mehrshahi et al., 2013). This study revealed similar access to chloroplast-localized substrates for ER-localized γ-tocopherol methyltransferase (γTMT) and α-carotene ε-ring hydroxylase LUTEIN DEFICIENT1 (LUT1). Based on their successful transorganellar complementation assay, the authors propose that an exchange of non-polar metabolites between plastids and endoplasmic reticulum occurs, most likely due to direct contacts between those organelles at PLAM sites according to the membrane hemifusion model (Mehrshahi et al., 2014). The hemifusion model postulates that the fused membrane of the ER and chloroplast outer envelope would consist of the inner leaflet of the ER and the plastid outer envelope membranes, two envelopes with similar compositions of non-polar metabolites (Mehrshahi et al., 2014). A membrane of this nature would create an easy path for chloroplast-to-ER signaling by non-polar metabolites.

### Chloroplast-Peroxisome Contacts

Peroxisomes are dynamic membrane-bound organelles of remarkable metabolic plasticity. They are found in all eukaryotic cells, and in plant cells are usually found in close association with mitochondria and chloroplasts (**Figure 3**). Peroxisomes are able to adjust their complement of enzymes in response to changes in environmental and developmental signals (reviewed in Goto-Yamada et al., 2015; Sandalio and Romero-Puertas, 2015). Peroxisomes were first recognized for their action in scavenging H_2_O_2_, however, it is clear that peroxisomes have much more extensive roles in cellular metabolism (Sandalio and Romero-Puertas, 2015). A subset of these reactions highlights the intimate metabolic coupling between chloroplasts and peroxisomes.

The photorespiratory pathway is the conversion of phosphoglycolate to CO_2_ and 3PGA, via a complex series of reactions that takes place across three separate subcellular compartments: chloroplasts, peroxisomes, and mitochondria (Foyer et al., 2009). Severe photorespiratory conditions initiate ROS-dependent lipid peroxidation in the chloroplast that leads to activation of the lipoxygenase (LOX)-mediated reaction, which is one of the starting points in the oxylipin metabolic pathway. LOX also initiates the synthesis of many cell constituents and signaling molecules, including jasmonates via oxo-phytodienoic acid (OPDA; Weber et al., 1997; Montillet et al., 2004). OPDA is then transported into the peroxisomes where two rounds of beta-oxidation serve to modify the fatty-acid side-chain of the ring. It is believed that the transport of OPDA is carried out by both active and passive transport (Leon, 2013).

Recent work has revealed that in addition to close metabolic coupling, peroxisomes and chloroplasts physically interact with each other in a light-dependent manner. Using the newly developed femtosecond laser technology and confocal laser scanning microscopy, Oikawa and colleagues demonstrated that peroxisomes adopted an elliptical shape to increase their surface area and more tightly adhered to chloroplasts in light (Oikawa et al., 2015). A force of 61 fN nm^-2^ was needed to disrupt the chloroplast–peroxisome interaction in the light compared to 23 fN nm^-2^ required to so do in the dark. Interestingly, these changes in peroxisome shape and location depended on photosynthesis, but were independent of photorespiration or the activity of photoreceptors, and the actin cytoskeleton negatively regulated the interaction between the chloroplasts and peroxisomes (Oikawa et al., 2015). The chloroplast–peroxisome physical interaction is consistent with reports from other systems that suggest direct interaction between organelles is necessary for metabolite exchange (Binns et al., 2006; de Brito and Scorrano, 2008). Thus the trafficking of compounds like OPDA during JA synthesis could potentially contribute to signaling between plastids and peroxisomes.

The *Arabidopsis snowy cotyledon3 (sco3-1)* mutant also provides support for signaling between chloroplasts and peroxisomes. In seedlings, the *sco3-1* mutation interrupted chloroplast biogenesis, decreased chlorophyll accumulation, and disrupted thylakoids and, as a consequence, photosynthesis (Albrecht et al., 2010). This mutation also resulted in photoinhibition in mature leaves under high CO_2_ concentrations. It is quite interesting that the SCO3 protein is initially targeted to peroxisomes. Also interesting is that loss of SCO3 function led to cytoskeletal defects, specifically affecting microtubules. Thus, both the cytoskeleton and peroxisomes are necessary for normal chloroplast development. Further investigation of SCO3 function will illuminate the process of communication between chloroplasts and peroxisomes.

### Chloroplast-Mitochondrion Contacts

Like chloroplasts, mitochondria are the end products of an endosymbiotic event, and they have also retained a portion of their ancestral genome (Woodson and Chory, 2008). Mitochondria-to-nucleus retrograde signaling is critical for coordinating expression of nuclear genes encoding mitochondrial proteins with expression of the mitochondrial genome (Woodson and Chory, 2008; Rhoads, 2011). Given their central roles in energy capture and utilization it is perhaps not surprising that chloroplasts and mitochondria exchange metabolites. Chloroplasts and mitochondria are also coupled by cellular redox status.

Mutant analyses have shed light on chloroplast-mitochondrion signaling. The expression of *alternative oxidase (AOX)*, a nucleus-encoded mitochondrial gene, seems to be regulated by chloroplasts as increased expression of *Arabidopsis* and soybean *AOX* has been observed upon high light treatment (Finnegan et al., 1997; Blanco et al., 2014). Intriguingly, the white leaves of the chloroplast ribosome-deficient barley mutant *albostrians* as well as photo-bleached leaves of wild type obtained upon treatment with norflurazon, displayed elevated levels of mitochondrial DNA and transcripts (Hedtke et al., 1999). Consistent with this observation, recent analyses of leaves with green/white variegation in 12 ornamental plants confirmed that chloroplast dysfunction leads to increased levels of mitochondrial DNA in white sectors (Toshoji et al., 2012). Communication between chloroplasts and mitochondria seems to be bidirectional as mutation of genes encoding mitochondrial proteins can have profound effects on chloroplasts (e.g., Xu et al., 2008; Burch-Smith et al., 2011).

Several possible routes for communication between chloroplasts and mitochondria are proposed. There is coordinated expression of nuclear genes encoding chloroplast and mitochondria proteins, and it is therefore likely that some of the chloroplast signaling to mitochondria is accomplished by modulating nuclear gene expression. Indeed, expression of the *MITOCHONDRIAL DYSFUNCTION STIMULON* (*MDS*) suite of mitochondria-associated genes was regulated in response to chloroplast perturbations that included increased ROS and NO production (Ng et al., 2014). These *MDS* genes carry a common regulatory motif in their promoters that mediates their induction in response to mitochondrial retrograde signals (De Clercq et al., 2013; Ng et al., 2013). Moreover, it was demonstrated that *Arabidopsis* ABI4 regulates both *Lhcb* and *AOX1A* genes, providing a molecular link for nucleus-coordinated chloroplast-mitochondria communication (Koussevitzky et al., 2007; Giraud et al., 2009). Analysis of the *Arabidopsis regulator of alternative oxidase 1* (*rao1)* mutant deficient in a nucleus-localized cyclin-dependent kinase E1 (CDKE1) that is a prerequisite for *AOX* induction, indicates it is another nucleus-localized sensor integrating mitochondrial and chloroplast retrograde signals (Blanco et al., 2014). Unlike wild type plants the *rao1(cdke1)* mutant was unable to induce *AOX1A* expression upon application of antimycin A or DCMU (that specifically targets chloroplasts). Further, the mutant displayed the *gun* phenotype upon induction of redox stress originating specifically from chloroplast photoelectron transport.

Another possible mechanism for chloroplast-to-mitochondrion signaling could involve dual targeting of proteins. The localization of proteins to multiple subcellular compartments is an ancient feature of land plants that can be observed in *Physcomitrella patens* (Xu et al., 2013b) and diatoms (Gile et al., 2015). In *Arabidopsis*, over 100 proteins are targeted to both chloroplasts and mitochondria (Carrie and Whelan, 2013). It is tempting to propose that, analogous to the situation where proteins have been shown to localize to both chloroplasts and nuclei, proteins may move from the chloroplast to the mitochondria. The regulated translocation of proteins from chloroplasts to mitochondria to modulate mitochondrial gene expression would mediate chloroplast signaling. Such translocation would be made much easier by direct contact between chloroplasts and mitochondria.

Third, there may be direct communication by physical interaction. In leaves, chloroplasts, mitochondria and peroxisomes have often been observed in close association, consistent with metabolic exchange among these organelles (**Figure 4**). It seems that the formation of this tri-organellar unit is regulated, with the chloroplast-peroxisome association being established first and then recruiting mitochondria (Oikawa et al., 2015). Application of the femtosecond laser pulses to chloroplast-mitochondria complexes in a variety of tissues should illuminate the biophysical characteristics of their association.

In all these instances of organelle interactions, it is apparent that more detailed analysis at the level of resolution provided by electron microscopy is needed. Such studies should incorporate state-of-the-art fixation techniques like tandem high-pressure freezing and freeze substitution in order to minimize artifact formation and to maintain intact the presumably delicate membrane extensions and contact sites. These techniques are becoming easier and less time-consuming (McDonald, 1999, 2014) and can easily be adopted for plant cell biology (Bobik et al., 2014). When coupled with fluorescence microscopy, this will be a powerful approach for ultrastructural interrogation, as exemplified by recent work from Caplan et al. (2015).

## Hormones and Reactive Molecules as Chloroplast Signals

As sessile organisms plants have evolved to cope with extreme environmental conditions and fluctuations. Some of the most common environmental challenges to plant survival include drought, flooding resulting in reduced oxygen availability, and temperature extremes. In addition, the photosynthetic machinery is sensitive to excessive light and such exposure results in oxidative stress at the cellular level. In addition to these abiotic stresses, pathogens pose a constant threat of disease. Plants have therefore evolved a complex suite of responses that are exquisitely fine-tuned to allow them to cope with these stresses. It is widely recognized that chloroplasts both sense and respond to environmental conditions. Indeed, the chloroplast-generated hormones SA, JA and ABA and other secondary messengers including ROS and reactive nitrogen species (RNS) as well as redox signals are critical components of the plant stress response. Therefore chloroplast signaling is indispensable for plant survival of abiotic and biotic stress. The roles of all these chloroplast-associated molecules in coping with biotic and abiotic stress have been extensively examined and several excellent reviews are available (Miller et al., 2008; Padmanabhan and Dinesh-Kumar, 2010; Kangasjarvi et al., 2012; Pfannschmidt and Yang, 2012; Trotta et al., 2014).

### Salicylic Acid

Salicylic acid is best known for its role plant–pathogen interactions and particularly in plant defense. However, SA also has roles in plant developmental processes including germination, root and shoot growth and senescence, and also functions in abiotic stress responses (Rivas-San Vicente and Plasencia, 2011). A phenolic compound, SA is largely the product of the isochorismate pathway in the chloroplasts. Pathogen infection induces the production of SA by chloroplasts mainly through the action of the chloroplast-localized ISOCHORISMATE SYNTHASE (ICS)1 enzyme although there may be some contribution by ICS2 (Wildermuth et al., 2001; Garcion et al., 2008). Analysis of the *ics1 ics2* double mutant has revealed that there are other cellular sources of SA (Garcion et al., 2008), most likely via phenylpropanoid metabolism in the cytoplasm (Vlot et al., 2009). SA conjugates with glucose or a methyl side-group are the commonly active forms of SA. Indeed, methyl salicylate is a critical mediator of systemic acquired resistance (SAR; Park et al., 2007), and it may also function in ecological defense signaling when it becomes airborne (Shulaev et al., 1997).

Many SA-mediated defense responses rely on the action of the transcriptional activator NON-EXPRESSOR OF PR1 (NPR1; Fu and Dong, 2013). NPR1 interacts with TGA transcription factors, and it is believed to act with them as co-activators of defense gene expression (Zhang et al., 1999; Zhou et al., 2000; Yan and Dong, 2014). The action of NPR1 is dependent on the cellular redox state. In the absence of SA, NPR1 oligomerizes in the cytoplasm, but on perception of SA, NPR1 is reduced and the oligomer disassembles into monomers that then relocate to the nucleus to modulate gene expression (Kinkema et al., 2000; Mou et al., 2003). The redox state of NPR1 is mediated by the glutathione and thioredoxin redox systems (Mhamdi et al., 2010; Han et al., 2013). In the nucleus, NPR3 and 4, two other SA-binding proteins (SABPs) that are closely related to NPR1, modulate NPR1’s activity (Fu et al., 2012). It should be noted that there are also NPR1-independent pathways that mediate SA signaling (Rairdan et al., 2001; Shah, 2003; Uquillas et al., 2004; Blanco et al., 2005). Downstream of NPR1 the SA signaling pathway is well understood (Spoel et al., 2003; Fu and Dong, 2013).

Salicylic acid is important for basal defense as well as for ETI (Alazem and Lin, 2015), and application of SA or overexpression of its biosynthesis genes leads to increased pathogen resistance (Malamy et al., 1990; Ryals et al., 1996; Mur et al., 2008; Shah, 2009; Coll et al., 2011). Chloroplast Ca^2+^ signals are induced in a stress-specific manner, and this response is mediated by the calcium-sensing receptor (CAS; Nomura et al., 2012). CAS mediates the Ca^2+^ signals in ETI and in response to the presence of highly conserved pathogen associate molecular patterns (PAMPS). CAS, and thus CA^2+^, regulates chloroplast SA biosynthesis and plants depleted of CAS failed to induce SA production in response to pathogen infection. In addition, expression of several nuclear defense-related genes was shown to be dependent on CAS, and the pattern of gene regulation was most similar to that observed in response to ^1^O_2._

Interestingly, exogenous application of SA induces closure of plasmodesmata (Wang et al., 2013b). This is mediated by PLASMODESMATA LOCALIZED PROTEIN (PDLP)5, and likely involves the action of a callose synthase (Lee et al., 2011; Wang et al., 2013b). Consistent with these findings, *Arabidopsis pdlp5* mutants have decreased resistance to bacterial pathogens. These results clearly demonstrate crosstalk between SA signaling and plasmodesmata, and illustrate how chloroplast signals can act to regulate intercellular trafficking via plasmodesmata (discussed below). It will be interesting to see if similar mechanisms are deployed for viral resistance since viruses use plasmodesmata for intercellular trafficking.

There has been some controversy over the SA-binding properties of NPR1. Binding assays using recombinant GST-NPR1 and tritiated-SA ([^3^H]-SA) suggested that NPR1 did not bind SA (Fu et al., 2012). In contrast, equilibrium dialysis experiments revealed that NPR1 bound SA with a K_D_ similar to that observed for other receptor-ligand interactions for plant hormones (Wu et al., 2012). Recent results confirm that SA can indeed bind NPR1 (Manohar et al., 2014). However, it is clear that NPR1 is not the only protein that binds SA. The metabolic enzymes catalase (Chen et al., 1993), ascorbate peroxidase (Durner and Klessig, 1995) and methyl salicylate esterase (SABP2; Forouhar et al., 2005) have been shown to bind SA. Recent work reveals that SA potentially has numerous targets in the cell including, not unexpectedly, numerous chloroplast proteins.

Many of these SABPs have been identified by high-throughput approaches (Moreau et al., 2013; Manohar et al., 2014). By probing *Arabidopsis* protein microarrays with 4-azido SA (azSA), an SA analog, the Popescu lab identified numerous chloroplast-localized proteins with roles in photosynthesis and oxidative phosphorylation as proteins interacting with AzSA (Moreau et al., 2013). Two other interesting candidate SABPs were also identified: thimet metalloendopeptidase At5g65620 (TOP1) and its homolog encoded by *At5g10540* (TOP2), both of unknown cellular function. TOP1-GFP fusions localized to chloroplasts and TOP2 is likely cytosolic. Interestingly, *top1 top2* double mutants had compromised ETI and PCD to bacterial pathogens. Further, TOP1 and TOP2 dimerize in an SA- and redox-dependent manner (Westlake et al., 2015). However, TOP1 and TOP2 have distinct responses to the reductant DTT, suggesting they have different activities *in planta*. A role for TOP1 and TOP2 in the oxidative stress response was demonstrated by treating various *top* mutants with methyl viologen, a potent inducer of oxidative stress, but this function is likely restricted to early in plant development. Two additional high throughput screens have recently identified another 100 candidate SABPs (Manohar et al., 2014). Of these, nine were already known SABPs and the SA binding of nine of new candidate SABPs was verified. Notably, four of the new SABPs have roles in redox regulation, reiterating the interaction between these two pathways.

### Jasmonates

Jasmonic acid is a lipid-derived hormone that is perhaps best known for its roles in insect herbivory and wounding, but also has roles in plant growth and development (Leon, 2013). The term jasmonates refers to a group of compounds that are derived from linoleic acid. JA is synthesized via the octadecanoid pathway and JA synthesis is initiated in the chloroplasts but is completed in the peroxisome. JA is then derivatized to yield a diverse array of metabolites that have different functions, ranging from storage to inactivation (reviewed in Leon, 2013; Wasternack and Hause, 2013). The most active of these compounds is a JA conjugate with isoleucine, *(+)-7-iso*-JA-Ile (Wasternack, 2014). JA biosynthesis and signaling pathways have been elucidated (Turner et al., 2002; Antico et al., 2012; Wasternack and Hause, 2013; Zhu, 2014). JA signaling in stress is closely liked with that of another hormone, ethylene (Kunkel and Brooks, 2002). Interestingly SA and JA/ET signaling are often antagonistic to each other (Robert-Seilaniantz et al., 2011). This highlights the crosstalk between chloroplast signals.

The final chloroplastic intermediate in JA-biosysnthesis is *cis*-(+)-12-oxophytodienoic acid (OPDA). OPDA is then translocated to the peroxisome, where its hydrocarbon chain is shortened by β-oxidation. It is not clear how OPDA is transported to the peroxisomes, but the process must be tightly regulated, given that OPDA itself is able to act as a signaling molecule. Indeed, OPDA has been implicated in tendril coiling (Blechert, 1999); in *Arabidopsis* seed germination (Dave et al., 2011); in tomato embryo development (Goetz et al., 2012), and fertility in *P. patens* (Stumpe et al., 2010). OPDA is also known to be an important signal for defense (Stintzi et al., 2001; Scalschi et al., 2015). Interestingly, *P. patens* does not make JA but instead uses OPDA for defense (Stumpe et al., 2010; Ponce De Leon et al., 2012). However, SA is used as a defense signal in this moss (Ponce De Leon et al., 2012). In the liverwort *Marchantia polymorpha* wounding induces OPDA production, and exogenous application limited *M. polymorpha* growth (Yamamoto et al., 2015). As in *P. patens*, JA was not detected in *M. polymorpha.* This finding was supported by OPDA regulating growth of *M. polymorpha* while JA could not. Thus, the production of lipid-derived signals by the chloroplast is an ancient feature of plants, and the production of JA may have developed more recently.

### Crosstalk of RNA Silencing and Chloroplast Hormones

There is also crosstalk between the chloroplast-derived phytohormones and the RNAi machinery of plant cells. Several studies have reported increased expression of *RNA-DEPENDENT RNA POLYMERASE1 (RDR1)* on exogenous application of SA (Xie et al., 2001; Yu et al., 2003; Hunter et al., 2013), JA (Pandey and Baldwin, 2007) and ABA (Hunter et al., 2013). RDR1 is known to have a role in antiviral RNA silencing, in the production and amplification of virus-derived siRNAs (Donaire et al., 2008; Wang et al., 2010). JA interaction with SA is mostly antagonistic, and there are few genes whose expression is induced by both hormones (Pieterse et al., 2009). The finding RDR1 can be induced by multiple hormones suggests that there is crosstalk between hormones and the RNA silencing machinery to mediate stress responses. However, RDR1 does not seem to have a role in drought resistance (Hunter et al., 2013), although other components of the RNAi components have been demonstrated to function in stress tolerance (Earley et al., 2010; Li et al., 2012; Westwood et al., 2013). Together, these findings extend the role of chloroplast signaling in the stress response.

### Abscisic Acid

Abscisic acid is one of the most important hormones mediating plant biotic and abiotic stress responses. ABA also has major roles in various plant physiological processes including stomatal movement and seed dormancy (Wensuo and Zhang, 2008; Rodriguez-Gacio Mdel et al., 2009; Kim et al., 2010). ABA is a sesquiterpenoid that is produced by the methylerythritol phosphate (MEP) pathway in plastids that produces carotenoids (Finkelstein, 2013). ABA levels in a cell are the result of both its synthesis and catabolism. Interestingly, several subcellular compartments are involved in ABA metabolism (Xu et al., 2013a). All the steps of the *de novo* ABA biosynthetic pathway occur in plastids except for the last two, which occur in the cytosol. The first committed step in ABA synthesis is the cleavage of the carotenoid xanthopyhll by 9-*cis*-epoxycarotenoid dioxygenase (NCED) to produce the C15 compound xanthoxin, that is transported from the plastids into the cytosol by an unknown mechanism, where it will be converted into ABA (Finkelstein, 2013). Once synthesized, ABA is transported from the sites of synthesis to sites action via the xylem and phloem and is thus made available to both roots and shoots. ABA catabolism occurs via one of two pathways: oxidation or conjugation to glucose to produce a glucosyl ester ABA-GE. ABA-GE is stored in the vacuole or ER until it mobilized under stress conditions. Because of its central roles in modulating responses to various stresses, ABA signaling is an attractive target for engineering plants with increased tolerance to those stresses.

The mechanisms governing perception of, and signaling by, ABA are being discovered (Yoshida et al., 2015). Members of the PYR/PYL/RCAR family of soluble proteins are ABA receptors (Ma et al., 2009; Park et al., 2009). This protein family has 14 members, almost all of which appear capable of forming an ABA-receptor complex that is able to activate the transcription of ABA-responsive genes (Kline et al., 2010). Downstream of the receptors, several kinases, including calcium dependent kinases (CDPKs), and phosphatases mediate ABA signaling, culminating in changes in nuclear gene expression (Finkelstein, 2013; Yoshida et al., 2015). The 26S proteasome is also important in mediating ABA signaling (Ludwikow, 2015).

Abscisic acid signaling is particularly important during drought, salinity and cold stress. During pathogen infection, ABA signaling is antagonistic to JA/Et signaling (Soosaar et al., 2005); and it can also antagonize SA signaling (Alazem and Lin, 2015). Indeed, crosstalk between SA, JA, and ABA signaling pathways during pathogen defense is well documented (Tuteja, 2007; Flors et al., 2008; Cao et al., 2011) and underscores the role of chloroplasts in integrating inputs for plant survival. Importantly, the H subunit of Mg chelatase was shown to be an ABA receptor, and moreover, the observations that ABA can repress *Lhcb* expression and the gun phenotype of the *abi4* mutant link this hormone to retrograde signaling (Shen et al., 2006; Koussevitzky et al., 2007).

### Reactive Oxygen Species

Reactive oxygen species are formed by the reduction of molecular oxygen and the term ROS includes superoxide (O_2_^⋅-^), hydroxyl, alkoxyl (^.^RO), and peroxyl radicals as well as non-radical molecules like hydrogen peroxide (H_2_O_2_) and singlet oxygen (^1^O_2_). It is well established that while large amounts of ROS are damaging, small amounts act as signaling molecules (Miller et al., 2008; Gill and Tuteja, 2010). In an effort to avoid the toxicity of ROS, plants have evolved multiple antioxidant systems. It is clear then that ROS signaling is complex and is often the outcome of the balance between production and scavenging.

In a typical plant cell ROS may be generated by a variety of subcellular compartments including chloroplasts, peroxisomes, mitochondria and the apoplast. In a photosynthesizing leaf most ROS is the product of the chloroplasts and peroxisomes with smaller contributions from mitochondria (Foyer and Noctor, 2005). During photosynthesis, in the reaction center of photosystem II (PSII), excited triplet center chlorophyll P680 interacts with oxygen to generate ^1^O_2_. The acceptor side of PSI produces superoxide and hydrogen peroxide as electrons are transferred from reduced ferredoxin to molecular oxygen. Photorespiration in the peroxisomes to recycle Rubisco that has reacted with oxygen is a major source of H_2_O_2_ (Yoshida and Noguchi, 2011).

Plastid-generated ROS molecules have been shown to act as signals that modulate expression of nuclear genes. Different ROS molecules have been shown to induce expression of distinct suites of genes (Desikan et al., 2001; Vandenabeele et al., 2004; Vanderauwera et al., 2005; Laloi et al., 2007; Li et al., 2011; Balazadeh et al., 2012; Mor et al., 2014). This has made it possible to draw distinctions between the various ROS initiating responses to different stresses, (e.g., Gadjev et al., 2006). By virtue of their nature, ROS are known to interact with a variety of biological molecules. For example, as a strong electrophile, ^1^O_2_ can react spontaneously with many classes of biological molecules including proteins, lipids and nucleic acids. However, the question remains whether they act directly or indirectly through modification of other biomolecules.

Hydrogen peroxide is the least reactive ROS, and at high light intensities up to 5% of chloroplast-generated H_2_O_2_ has been detected outside the organelle (Mubarakshina et al., 2010). H_2_O_2_ may exit the chloroplasts via aquaporins (Bienert et al., 2007). Recently, H_2_O_2_ has also been shown to relocate from chloroplasts to the nucleus via stromules (Caplan et al., 2015). Indeed, chloroplast ROS production induces stromule formation (Brunkard et al., 2015b). These findings suggest that H_2_O_2_ may act directly on its targets to regulate their expression or behavior.

In contrast, ^1^O_2_ is highly reactive, and, ^1^O_2_ is involved in signaling pathways leading to cell death or to acclimation (Wagner et al., 2004; Ledford et al., 2007). Yet, because of its very high reactivity and therefore short lifetime *in vivo*, ^1^O_2_ is not considered to be a molecule directly involved in chloroplast-to-nucleus signaling. Studies in the *Arabidopsis fluorescent (flu)* mutant have shed light on ^1^O_2_ signaling (Kim and Apel, 2013). The *flu* mutant accumulates protochlorophyllide and when plants are exposed to light they generate large amounts of ^1^O_2_ (Meskauskiene et al., 2001). Increased ^1^O_2_ production in *flu* chloroplasts was associated with induction of stress responses including dramatic changes in nuclear gene expression and enhanced accumulation of the stress hormones, SA, Et, and the oxylipins OPDA and JA (op den Camp et al., 2003). Interestingly, these ^1^O_2_-induced changes were dependent on the chloroplastic EXECUTER1 (EX1) and EX2 proteins (Kim et al., 2012). To date, two direct protein targets of ^1^O_2_ have been identified: one is β-carotene and the other is the D1 protein of PSII (Kim and Apel, 2013). Reaction of ^1^O_2_ with β-carotene produces β-cyclocitral, shown to be involved in retrograde signaling (Ramel et al., 2012; see above). D1 is potent scavenger of ^1^O_2_, and this activity leads to the destruction of D1 by protease activity. However, to date no biological activity for these D1 fragments in plants has been reported.

Plants possess several enzymes including catalase and ascorbate peroxidase that detoxify ROS (Miller et al., 2008). In addition plants have evolved antioxidant systems that allow them to not only cope with ROS production during stress but also transduce ROS signals. Such systems include tocopherols (vitamin E), ascorbate (vitamin C), and glutathione (a tripeptide thiol) and they all associate with chloroplasts. Glutathione is the most important antioxidant in plants (Zechmann, 2014) and it can act on ROS directly or through the ascorbate-glutathione system. Glutathione synthesis is initiated in chloroplasts (Wachter et al., 2005). The importance of glutathione highlights the importance of thiol-disulfide reactive proteins as antioxidants (Foyer and Noctor, 2003). Proteins including thioredxoxins, glutaredoxins, glutathione peroxidases are found in chloroplasts as well as other subcellular compartments, and these proteins convey the redox state of chloroplasts to the rest of the plant cell. The involvement of redox signaling in mediating plant stress responses are extensively documented (Foyer et al., 2009).

### Nitric Oxide and Reactive Nitrogen Species

Nitric oxide (NO) is a small gaseous molecule whose role in signaling in plant and non-plant systems is well established. While NO is an important second messenger in plants, major aspects of NO synthesis and action remain undiscovered. NO has critical roles in normal plant physiology including seed dormancy, germination, the floral transition, and during stress responses (Wendehenne et al., 2004; Moreau et al., 2008; Sanz et al., 2015). In plants NO is synthesized in chloroplasts (Mandal et al., 2012; Tewari et al., 2013), and synthesis is closely linked to lipid metabolism, specifically that of oleic acid (Mandal et al., 2012).

In animals NO is synthesized by NITRIC OXIDE SYNTHASEs (NOSs) that oxidize L-arginine to NO (Stuehr, 2004). Curiously, NOS-like activity has long been described in plants but the identity of this enzyme remains elusive (Besson-Bard et al., 2008). In plants, nitrate reductase can reduce nitrate to NO (Desikan et al., 2002). Another enzyme implicated in NO synthesis is AtNOA1 (formerly AtNOS1). However, AtNOA1 is a GTPase that binds ribosomes, and it lacks NOS activity (Moreau et al., 2008). Nonetheless, *atnoa1* mutants displayed reduce levels of NO (Guo et al., 2003).

One of the primary mechanisms through which NO exerts its effects is by direct regulation of protein activity through nitrosylation of cysteine residues in a redox-dependent manner (Mengel et al., 2013). Nitration of tyrosine residues also occurs, and it is mediated by the peroxynitrite (ONOO^-^) RNS formed on reaction of NO with O_2_ (Corpas et al., 2013). Tyrosine nitration is viewed as a hallmark of plants under a variety of biotic and abiotic stresses (Corpas et al., 2013). While proteomic analyses have identified only relatively few nitrosylated proteins in *Arabidopsis* (Lindermayr et al., 2005; Romero-Puertas et al., 2008; Ortega-Galisteo et al., 2012; Camejo et al., 2013; Puyaubert et al., 2014), a recent computational approach identified more than 16, 000 potential protein target for nitrosylation (Chaki et al., 2014). Refined versions of this program should be very useful in streamlining investigation of NO function and effects in signaling.

There is substantial crosstalk between ROS and RNS signaling pathways (Lounifi et al., 2013). Indeed, even small amount of NO had profound effects on chloroplast PET and redox state (Vladkova et al., 2011; Misra et al., 2014). It is therefore not surprising that NO is important for plant defense (Zaninotto et al., 2006; Bellin et al., 2013). Further, NO signaling interacts with SA and JA signaling pathways (Wendehenne et al., 2004; Zhou et al., 2015). NO production has been proposed as a potential target for engineering plant fitness (Foresi et al., 2015). However, this approach should be carefully considered given the potentially deleterious effects of runaway NO production and the extensive crosstalk between NO and other stress signaling pathways.

### Chloroplasts as Targets of Pathogen Effectors

The previous sections have highlighted the role of several chloroplast products in stress tolerance and plant defense. That chloroplasts have critical roles in pathogen defense is supported by the identification of numerous chloroplast proteins as direct targets of pathogen effectors (**Table 1**). Several of these targets are components of PET, suggesting that photosynthesis and chloroplast function are important for defense against pathogens. A series of experiments aimed at addressing the role of chloroplasts beyond phytohormone production in plant defense has been reported. VIGS of two genes encoding components of the PSII oxygen-evolving complex, *PsbQ* and *PsbO*, resulted in a three to four-fold increase in the number of Turnip mosaic virus (TuMV) infection foci (Manfre et al., 2011). A similar increase was observed when the chloroplast protease FtsH or Rubisco were depleted by VIGS (Manfre et al., 2011). These findings were further supported by the observation that treatment with lincomycin, and low-light conditions led to increased rates of TuMV infection. Importantly, it was demonstrated that these effects were independent of SA, as lincomycin-treated plants induced SA production to the same levels on TuMV infection as observed in non-treated controls. Given that TuMV replicates in association with the chloroplast outer envelope (Wei et al., 2010), it is perhaps not surprising that perturbing chloroplast function results in increased viral infection. However, chloroplast proteins are targets for other viruses, e.g., Tobacco mosaic virus (TMV), which are not known to replicate in association with chloroplast membranes. Interestingly, PsbO has been shown to interact the helicase domain of the TMV replicase, and VIGS of *PsbO* lead to a 10-fold increase in TMV infection in *N. benthamiana* (Abbink et al., 2002).

**Table 1:** Chloroplast proteins targeted by pathogen effectors.

Pathogen	Pathogen protein	Target	Reference
**Viruses**			
Alfalfa mosaic virus	Coat protein	PsbP	Balasubramaniam et al., 2014
Tobacco mosaic virus	Replicase (helicase)	NRIP1	Caplan et al., 2008
	Replicase	PsbO	Abbink et al., 2002
		AtpC	Bhat et al., 2013
		Rca	Bhat et al., 2013
Tomato mosaic virus	Movement protein	RbcS	Zhao et al., 2013
Plum pox virus	CI	PSI-K	Jimenez et al., 2006
Potato virus Y	HC-Pro	MinD	Jin et al., 2007
		XDS	Li et al., 2015
Soybean mosaic virus	P1	Rieske Fe/S	Shi et al., 2007
Sugarcane mosaic virus	HC-Pro	Ferredoxin	Cheng et al., 2008
Turnip mosaic virus	P3	Rubisco	Lin et al., 2011
Alternanthera mosaic	TGB3	PsbO	Jang et al., 2013
virus			
**Bacteria**			
*Pseudomonas syringae* pv.	HopI1	Cytosolic	Jelenska et al., 2007
Tomato DC3000		Hsp70^∗^	Jelenska et al., 2010
	HopN1	PsbQ	Rodriguez-Herva et al., 2012
	HopU1	RNA binding proteins (CP-RBPs)	Fu et al., 2007
	HopK1	Unknown	Li et al., 2014
*P. syringae* pv pisi	AvrRps4	Unknown	Li et al., 2014
**Fungi**			
Melampsora larici-populina	MLP10772	Unknown	Petre et al., 2015
(rust fungus)	MLP124111		

## Chloroplasts and Intercellular Signaling

The engulfment of cyanobacteria and subsequent evolution of the cellulosic cell wall surrounding the new plant cell likely created a physical barrier that hampered cell-to-cell communication and further development of multicellular, three-dimensional plants. It is important to highlight that modern chloroplasts are a sister group to the filament and heterocyst-forming cyanobacteria *Nostoc* and *Anabaena* (Falcon et al., 2010). Intercellular movement of molecules between the cells of *Anabaena cylindrica* filaments has been demonstrated (Mullineaux et al., 2008). Heterocysts are specialized cells that fix N_2_ and lack O_2_-evolving PSII and typically they have thick cell walls with limited permeability to gases. Interestingly, heterocysts are connected to vegetative cells by microplasmodesmata (Falcon et al., 2010; Padmanabhan and Dinesh-Kumar, 2010). Thus, we propose that chloroplasts, as the successors of these ancient cyanobacteria, participate in regulation of the cell-to-cell connectivity of modern plant cells by regulating plasmodesmata biogenesis in contemporary plants. This hypothesis is supported by a number of reports showing that mutants with defective chloroplasts display aberrant trafficking and altered expression of cell wall-related genes (see below). The relationship between chloroplasts and plasmodesmata and thus cell wall becomes more palpable when one considers sugar transport from source to sink tissues through plasmodesmata.

### Chloroplast Signaling and Symplasmic Transport

Communication between plant cells is enhanced by the presence of plasmodesmata, specialized channels traversing the cell wall between adjacent cells. Even though discovered more than 100 years ago, the structure and regulation of plasmodesmata are poorly understood. In contrast, recent advances in plant biology and genetics have helped reveal much about their function. Plasmodesmata are indispensable for proper plant development as they provide a route for exchange of metabolites including water, ions and photoassimilates, as well as information encoded by hormones, nucleic acids and proteins including transcription factors (reviewed in Gallagher et al., 2014; Brunkard et al., 2015a; Heinlein, 2015; Jackson, 2015). Plant viruses have evolved to take advantage of the intercellular connectivity afforded by plasmodesmata, and viruses use these channels for cell-to-cell spread during infections of their plant hosts. Recent studies on grafted plants resulted in a tantalizing hypothesis that plasmodesmata facilitate the movement of the entire chloroplast genomes between neighboring cells (Stegemann and Bock, 2009; Stegemann et al., 2012; Thyssen et al., 2012). However, the dimensions of plasmodesmata would constrain movement of intact organelles, and whether the translocated genomes use intact chloroplasts as vehicles or are transported through plasmodesmata as “naked” but specifically folded DNA remains to be elucidated.

Interestingly, genetic screens aimed at isolating genes regulating plasmodesmata have identified genes involved in the biogenesis and/or functioning of chloroplasts. The first such reported mutant was maize *sucrose export defective1 (sxd1;* Russin et al., 1996). The *sxd1* mutants failed to export photosynthate from sites of photosynthesis and have reduced intercellular transport due to accumulation of callose at plasmodesmata located at the bundle-sheath and vascular parenchyma interfaces (Botha et al., 2000). Callose is a polymer composed of β-1,3-linked glucose residues and callose accumulation at the necks of plasmodesmata is a major strategy for limiting plasmodesmal trafficking (De Storme and Geelen, 2014). Notably, despite accumulating sugars and starch in source tissues, *sxd1* does not repress photosynthesis (Provencher et al., 2001). *SXD1* is the maize ortholog of *Arabidopsis VTE1*, encoding a chloroplast TC required for the production of the antioxidant vitamin E (Provencher et al., 2001; Porfirova et al., 2002). The *sxd1* mutant provided the first clue that chloroplast redox state may influence plasmodesmata. However, under optimal growth conditions *vte1* mutants were indistinguishable from wild-type plants, although they were more sensitive to photooxidative stress (Porfirova et al., 2002). *Arabidopsis* mutants with defects in tocopherol metabolism should be carefully examined for plasmodesmata-related changes in intercellular trafficking.

A genetic screen for *Arabidopsis thaliana* mutants with altered plasmodesmal function identified the *gfp arrested trafficking (gat)* mutants (Benitez-Alfonso et al., 2009). In these mutants GFP synthesized in the companion cells of the phloem failed to move via the plasmodesmata into the surrounding tissues as occurred in wild-type tissues; that is, *gat* mutants have decreased intercellular trafficking. The *gat1, 2, 4*, and *5* mutations are all seedling lethal, with development ceasing about 10 days after germination. *GAT1* encodes a plastid-localized thioredoxin-m3 (TRX-m3), and *gat1* roots accumulate higher levels of ROS than do wild-type roots. In addition, callose was found to accumulate at plasmodesmata in *gat1* roots. Similar observations have been made for the other *gat* mutants (Benitez-Alfonso and Jackson, 2009). Notably, approximately five percent of plasmodesmata in *gat1* seedling roots are occluded by electron-dense material (Benitez-Alfonso et al., 2009). Overexpression of *GAT1* results in the reciprocal phenotype of increased intercellular transport. Thus, like SXD1/VTE1, GAT1 likely functions in redox homeostasis involving the chloroplasts and perturbation of plastid redox state results in altered plasmodesmata. In addition, it has been proposed that altered metabolic flux in the *gat1* mutant may alter the redox state of TRX-m3 and ultimately influence plasmodesmata function (Benitez-Alfonso et al., 2009).

The Zambryski lab conducted a separate screen for *Arabidopsis* mutants with altered plasmodesmata-mediated intercellular trafficking (Kim et al., 2002; Burch-Smith and Zambryski, 2012). Several *increased size exclusion limit (ise)* mutants were identified by screening embryonically lethal mutants and monitoring their ability to traffic fluorescent dyes between cells. *ISE1* and *ISE2* have been mapped and cloned (Kobayashi et al., 2007; Stonebloom et al., 2009). In addition to increased plasmodesmal trafficking *ise1* and *ise2* embryos also contain increased numbers of plasmodesmata with multiple branches (Burch-Smith and Zambryski, 2010). Interestingly, nuclear *ISE2* encodes a chloroplast-localized DEVH-type RNA helicase, (Burch-Smith et al., 2011) while *ISE1* encodes a mitochondrial DEAD-box RNA helicase (Stonebloom et al., 2009). Further analyses in *Arabidopsis* and *N. benthamiana* revealed that loss of ISE1 and/or ISE2 leads to defective chloroplasts and ultimately leaves become yellow (Burch-Smith and Zambryski, 2010; Burch-Smith et al., 2011; Stonebloom et al., 2012). Gene expression analyses on *ise1* and *ise2* embryos by tiling microarrays revealed that the largest class of nuclear genes affected in both mutants, representing ∼20% of the total, encode chloroplast-localized proteins (Burch-Smith et al., 2011). Importantly, genes encoding products for chlorophyll biosynthesis, photosynthetic light-harvesting reactions, and the Calvin cycle for carbon fixation were all affected in both mutants. Thus, the overlapping plasmodesmatal phenotypes of the *ise1* and *ise2* mutants are likely due to defective chloroplasts.

The changes in plasmodesmata and intercellular trafficking are likely mediated by altered redox signaling from chloroplasts and mitochondria. Using redox-sensitive fluorescent reporters the redox state of chloroplasts depleted of ISE1 or ISE2 was measured (Stonebloom et al., 2012). Significantly, chloroplasts in *ISE1*- or *ISE2*-silenced *N. benthamiana* leaves were more reduced than in non-silenced control leaves. These results are consistent those from the *gat1* mutant (Benitez-Alfonso et al., 2009). Thus, oxidized chloroplasts are associated with decreased intercellular trafficking while reduced chloroplasts increase plasmodesmata-mediated trafficking. Further experiments focused on identifying the redox pathways and signal transduction involved in regulating plasmodesmata will yield important insight into how the physiological state of the chloroplasts is communicated to the rest of the plant.

### Redox Signals Travel between Cells

Recently the involvement of chloroplasts in remote control of nucleus-localized alternative splicing was reported. Specifically, the light-regulated redox state of plastoquinone is the source of a chloroplast-generated signal able to control alternative splicing (Petrillo et al., 2014). In the presence of a reduced or oxidized plastoquinone pool, the alternative splicing of selected nuclear genes is promoted or inhibited, respectively. Importantly, even though photosynthetic tissues generated it, the putative signal is able to travel to roots to control alternative splicing. Thus chloroplast redox signals can act non-cell-autonomously. The redox pathway leading to defective alternative splicing has not been identified; however, the redox-regulated protein kinases of chloroplasts are proposed to contribute to the signaling machinery through a process of phosphorylation of some other proteins (Petrillo et al., 2014). This result is consistent with the observation that an *Arabidopsis* plasma-membrane-localized thioredoxin, Trx h9 moved intercellularly (Meng et al., 2010), presumably via plasmodesmata. The potential for intercellular trafficking by redox signals greatly extends the reach of chloroplasts in signaling.

The question remains: how could chloroplasts regulate plasmodesmata and intercellular trafficking? It is possible that as-yet-unidentified chloroplasts signals could directly target plasmodesmata. Indeed, chloroplasts are often observed in the vicinity of plasmodesmata, and regardless of the reasons for this close proximity, this means signals would not have to travel across long distances to act (**Figure 4**). Studies in the algae *Chara australis* also suggest a relationship between chloroplast and cell wall structure (Foissner et al., 2015). It is also possible that chloroplast-to-nucelus retrograde signaling may be involved in regulating events at the cell wall. Indeed, disruption of chloroplast function often results in significant changes of expression of genes involved in cell wall synthesis and modification (Burch-Smith et al., 2011). Similarly, expression of genes with functions related to the cell wall was down regulated in plants accumulating β-cyclocitral upon high light treatment (Ramel et al., 2012). It is also perhaps significant that three of the 39 genes identified as the core response module of chloroplast retrograde signaling encode proteins involved in synthesizing or modifying cell walls (Glasser et al., 2014). Future experiments should determine the pathways used for chloroplast-to-plasmodesmata signaling.

## Chloroplast Signaling in Ecosystem Functioning – the Power of Scent

In addition to being the center for production of oxygen and sugars, chloroplasts are also the source of plethora of secondary metabolites. Chloroplast-produced biogenic volatile organic compounds (BVOCs) seem to represent a fascinating signaling system that enables a plant to communicate with not only itself (e.g., propagation of a systemic response), and with the entire ecosystem that includes plant–plant, plant–animal/insect interaction, but could potentially also impact atmospheric chemistry (Atkinson, 2000; Seybold, 2006; Heil and Silva Bueno, 2007; Heil, 2010).

Volatiles directly produced in chloroplasts include isoprene, monoterpenes, diterpenes, hemiterpenes and volatile carotenoid derivatives, all synthesized by the MEP pathway. Also generated by the chloroplasts are green leaf volatiles (GLVs), synthesized in the LOX-pathway that consists of C6 and C9 aldehydes, alcohols and their acetate esters, that are responsible for the ‘mowed grass odor’ (Dudareva et al., 2013). These volatiles have important physiological roles, for example, stabilization of thylakoid membranes, reduction of ROS, as well as an ecological function, emphasizing an often underestimated contribution of chloroplasts/plastids to fine regulation of the ecosystem (Velikova et al., 2012). It is estimated that each year about 500 Tg C of isoprene is emitted to the atmosphere. This significant amount has some consequences as the highly reactive isoprene molecule can oxidize OH groups to yield peroxyl radicals that in turn can convert NO into NO_2_, producing O_3_ (Thompson, 1992; Carter, 1994; Atkinson, 2000). Therefore isoprene as a reactive molecule plays a crucial role in establishing the content of atmospheric greenhouse gasses and pollutants (ozone, methane, secondary organic aerosols, etc.; Harrison et al., 2013).

Chloroplast-produced BVOCs have other ecological functions that are a consequence of their signaling propensity. One of these is the so-called priming effect that leads to enhanced and more effective defense response to pathogen or insect attack in plants pretreated with volatiles (Engelberth, 2004). Although they are released continuously, there is enhanced emission of volatiles upon biotic or abiotic stresses including drought, high light, parasite or herbivore activity (wounding), resulting in so-called HIPVs (herbivore induced plant volatiles) emissions that include monoterpenes and GLVs (Brilli et al., 2009; Bonaventure and Baldwin, 2010; Dicke and Baldwin, 2010; Niinemets, 2010, 2013; War et al., 2011; Heil, 2014). Leaves of attacked plants emit volatiles that can prime defense responses in intact leaves of the same plant as well as on neighboring plants. Moreover, as demonstrated in maize, the intact plants exposed to HIPVs (termed receiver plants) are able to store the information conveyed by volatiles and recall it upon herbivore attack to activate defense genes encoding proteinase inhibitors (Ali et al., 2013).

The molecular mechanism of volatile signaling is far from understood, however, an attractive scenario of volatile-driven epigenetic modification through DNA methylation has been demonstrated as a very reliable strategy for priming defense induction. Moreover, since the JA-signaling pathway is triggered upon herbivore attack (wounding), it is expected that induced JA formation as a downstream effect would be relevant to the HIPV-primed defense responses (Ali et al., 2013). However, some of the most important questions concern the perception and the very first steps in volatiles signaling in receiver plants. To this end it is proposed that volatiles, as hydrophobic compounds, could diffuse through the outer, lipophilic leaf layer (cuticle) or enter through stomata (Baldwin et al., 2006). Indeed, strong membrane depolarization as well as calcium influx were observed upon treatment of tomato plants with GLVs, two phenomena often associated with a signal transduction cascade in other systems (Zebelo et al., 2012). Rearrangements of cytoskeleton and expression of selected genes encoding, among others, protein kinases and transcription factors as well as changes in stomatal aperture were observed upon treatment with the monoterpenes camphor and menthol (Kriegs et al., 2010). However, the concentrations used in the assay are inconsistent with physiological levels, thus the observed effects are questionable. Elucidating the impact of BVOCs on gene expression and impacts on general cell biology are a likely future direction of this field.

Another intriguing aspect of chloroplast originating volatiles is that their release from attacked plants can serve as attractants of the offending herbivore’s natural biological enemies, a phenomenon known as tritrophic chemical communication or tritrophic interaction (Heil, 2008; Mooney et al., 2012; Scala, 2013). Importantly, some volatiles also have allelopathic and insecticidal properties that could be deterrents for oviposition or can attract pollinators and/or seeds dispersers (Schulz et al., 2007; McCallum et al., 2011; Rodriguez et al., 2011; Reis et al., 2014). Together, these reports shed new light on chloroplasts as factories that generate volatile compounds that are important not only from the perspective of a single plant, but also the entire ecosystem. As new strategies for chloroplast biotechnology are deployed, it will be important to examine their effects on these volatiles so that susceptibility to herbivore predation and pathogen ingress are not inadvertently introduced through unintended modification of volatile production and perception.

## Conclusion and Future Directions

The era of transcriptomics initiated by the microarray platform and continued by Next-generation sequencing technology, together with metabolomics and proteomic approach provided by mass-spectrometry, established new possibilities and new directions in addressing questions in biology. Inevitably, due to these two strategies contemporary plant biology is progressing toward a more holistic view, which translates into studying not a single protein *per se*, but a single protein in the context of an entire cell, tissue, organ, individual plant and ultimately the entire ecosystem biology. Current progress in studying chloroplast biology shows their function goes far beyond photosynthesis and includes all aspects of plant biology, making these organelles an integral part and a full-fledged player in the cell. Understanding the signaling pathways chloroplasts and other plastids use to communicate with the rest of the plant cell under normal and stress conditions will provide new ideas for developing powerful strategies for engineering improved plants. Moreover, a thorough knowledge of chloroplast signaling will also allow the design of rational approaches that could minimize if not avoid unintended crosstalk and undesirable outcomes.

## Conflict of Interest Statement

The authors declare that the research was conducted in the absence of any commercial or financial relationships that could be construed as a potential conflict of interest.
